# Supplementary material for: Quantum control of ion-atom collisions beyond the ultracold regime
Source: Sci Adv. 2025 Feb 5;11(6):eadr8256. doi: 10.1126/sciadv.adr8256 (PMC11800771; doi:10.1126/sciadv.adr8256)
Supplement: Supplementary file 1 — Figs. S1 to S3 Legend data S1 [file sciadv.adr8256_sm.pdf]

Science Advances
AAAS

Supplementary Materials for

Quantum control of ion-atom collisions beyond the ultracold regime

Maks Z. Walewski *et al.*

Corresponding author: Maks Z. Walewski, mz.walewski@uw.edu.pl; Michał Tomza, michal.tomza@fuw.edu.pl

Sci. Adv. **11**, eadr8256 (2025)
DOI: 10.1126/sciadv.adr8256

The PDF file includes:

Figs. S1 to S3
Legend data S1

Other Supplementary Material for this manuscript includes the following:

Data S1

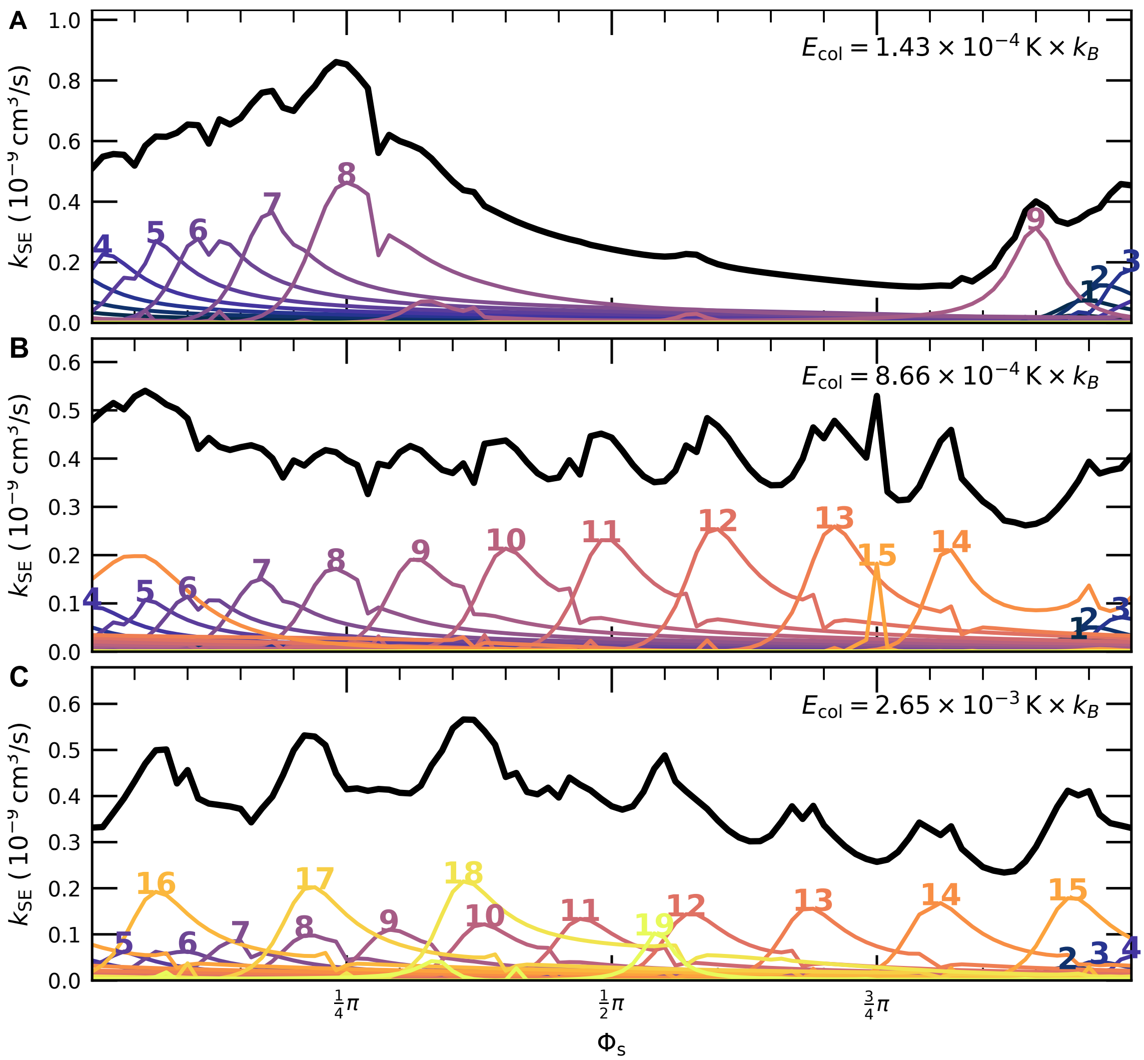

Figure S1: **Partial-wave contributions to the energy-resolved rate coefficients**. Spin-exchange rate coefficients ($|1,-1\rangle\,|\uparrow\rangle \rightarrow |1,0\rangle\,|\downarrow\rangle$) at fixed collision energies are plotted as a function of the singlet phase Φ_s for all relevant partial waves. The phase difference is fixed to $\Delta\Phi = 0.2\pi$ coming from the fit to the experimental data. At low collision energies (**A**), the relatively small number of partial waves introduces a large variation of the total inelastic rate coefficient (black line). At intermediate energies (**B**), most typical for $T_{exp} \approx 0.5\,\mathrm{mK}$, the variation is suppressed as more partial waves cover the full range of Φ_s from 0 to π. At higher collision energies (**C**), any possible enhancement comes from the different number of peaks from individual partial waves contributing to the total rate for different values of Φ_s. Notably, positions of the peaks for individual partial waves remain stable over a broad energy range in the millikelvin regime.

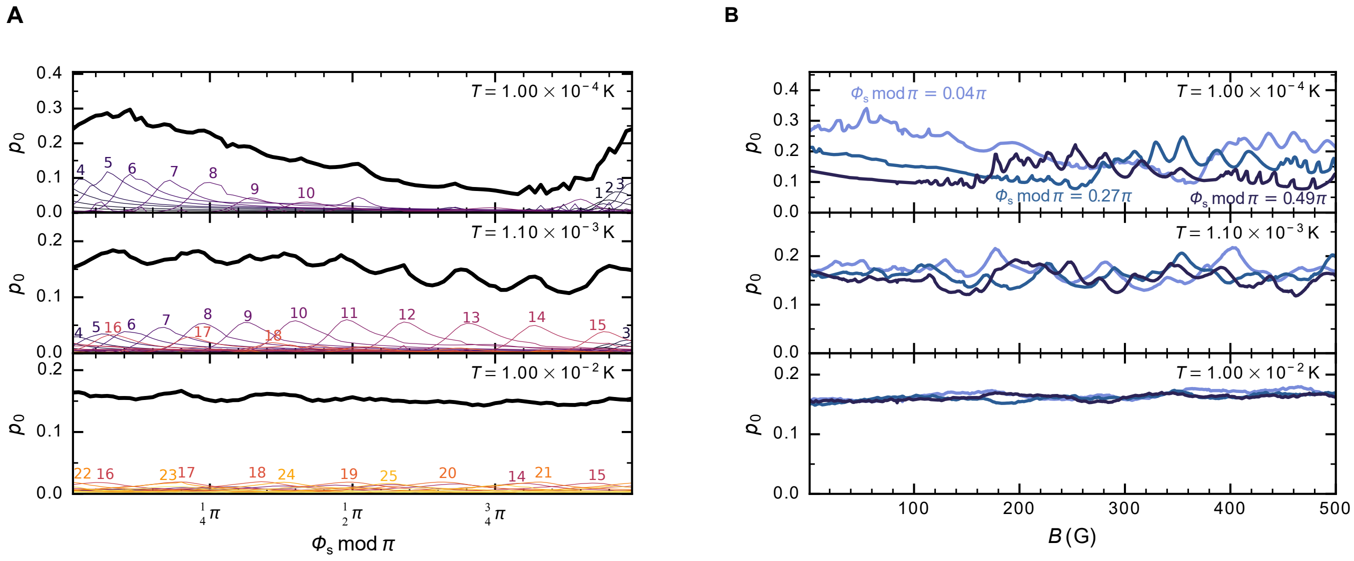

Figure S2: **Feshbach resonances in the multiple-partial-wave regime**. Calculated short-range probability p_0 of a spin flip of the $^{88}Sr^+$ ion, prepared in the $|\uparrow\rangle$ spin state, after a collision with an ^{87}Rb atom in the $|1, -1\rangle$ spin state, assuming the fitted value of phase difference $\Delta\Phi_{\text{fit}} = 0.2\pi$ at three different temperatures (0.1, 1, and 10 mK). (**A**) The probability calculated at $B = 2.97$ G as a function of Φ_s is indicated by the black solid line at each temperature, and the partial-wave contributions are labelled by the value of L. (**B**) The probability calculated as a function of magnetic field for three different values of $\Phi_s \bmod \pi = 0.04\pi, 0.27\pi, 0.49\pi$ at each temperature.

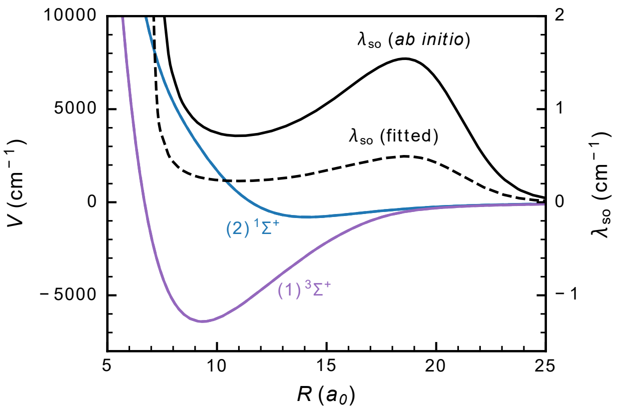

Figure S3: **Interaction potentials**. Calculated (*ab initio*) singlet $(2)\,^1\Sigma^+$ and triplet $(1)\,^3\Sigma^+$ potential energy curves are indicated by the blue and purple solid lines, respectively. Our *ab initio* second-order spin-orbit coefficient λ_{so} is drawn as the black solid line, and λ_{so} multiplied by the fitted value of $c_{\mathrm{so}} = 0.32$ is indicated by the black dashed line.

Data S1: Source data for main text figures and the interaction potentials. The source data for the appropriate subplots in Figs. 1-3 is provided as separate sheets in the .xlsx file. In the final sheet, our *ab initio* (2) $^1\Sigma^+$ and (1) $^3\Sigma^+$ potential energy curves are provided in the numerical form for the internuclear distance $R < 50\,a_0$ together with the van der Waals coefficients and parameters needed for the RKHS interpolation and extrapolation. We also provide our calculated second-order spin-orbit coupling $\lambda_{\mathrm{so}}(R)$ and the short-range scaling factors applied to the ab initio potential energy curves to obtain the given values of semiclassical phases $\Phi_{\mathrm{s}}, \Phi_{\mathrm{t}}$.
